# Advanced breast diffusion-weighted imaging: what are the next steps? A proposal from the EUSOBI International Breast Diffusion-weighted Imaging working group

**DOI:** 10.1007/s00330-024-11010-0

**Published:** 2024-10-08

**Authors:** Maya Honda, Eric E. Sigmund, Denis Le Bihan, Katja Pinker, Paola Clauser, Dimitrios Karampinos, Savannah C. Partridge, Eva Fallenberg, Laura Martincich, Pascal Baltzer, Ritse M. Mann, Julia Camps-Herrero, Mami Iima, on behalf of the EUSOBI International Breast Diffusion-Weighted Imaging working group, Maya Honda, Eric E. Sigmund, Katja Pinker, Paola Clauser, Dimitrios Karampinos, Savannah C. Partridge, Eva Fallenberg, Laura Martincich, Pascal Baltzer, Ritse M. Mann, Julia Camps-Herrero, Mami Iima, Denis Le Bihan

**Affiliations:** 1 Department of Diagnostic Imaging and Nuclear Medicine, Kyoto University Graduate School of Medicine, Kyoto, Japan; 2 Department of Diagnostic Radiology, Kansai Electric Power Hospital, Osaka, Japan; 3 Department of Radiology, NYU Langone Health, 6, 60 1st Avenue, New York, NY 10016 USA; 4 NeuroSpin/Joliot, CEA-Saclay Center, Paris-Saclay University, Gif-sur-Yvette, France; 5 Human Brain Research Center, Kyoto University Graduate School of Medicine, Kyoto, Japan; 6 National Institute for Physiological Sciences, Okazaki, Japan; 7 Department of Radiology, Breast Imaging Division, Columbia University Vagelos College of Physicians and Surgeons, New York, NY USA; 8 Department of Biomedical Imaging and Image-guided Therapy, Division of Molecular and Structural Preclinical Imaging, Medical University of Vienna/Vienna General Hospital, Wien, Austria; 9 Department of Diagnostic and Interventional Radiology, Technical University of Munich, Munich, Germany; 10 Department of Radiology, University of Washington School of Medicine, Seattle, WA USA; 11 Unit of Radiodiagnostics, Ospedale Cardinal G. Massaia -ASL AT, Via Conte Verde 125, 14100 Asti, Italy; 12 Department of Diagnostic Imaging, Radboud University Medical Centre, Nijmegen, Netherlands; 13 Ribera Salud Hospitals, Valencia, Spain; 14 Department of Fundamental Development for Advanced Low Invasive Diagnostic Imaging, Nagoya University Graduate School of Medicine, Nagoya, Japan

**Keywords:** Breast neoplasms, Diffusion magnetic resonance imaging, Surveys and questionnaires

## Abstract

**Objectives:**

This study by the EUSOBI International Breast Diffusion-weighted Imaging (DWI) working group aimed to evaluate the current and future applications of advanced DWI in breast imaging.

**Methods:**

A literature search and a comprehensive survey of EUSOBI members to explore the clinical use and potential of advanced DWI techniques and a literature search were involved. Advanced DWI approaches such as intravoxel incoherent motion (IVIM), diffusion kurtosis imaging (DKI), and diffusion tensor imaging (DTI) were assessed for their current status and challenges in clinical implementation.

**Results:**

Although a literature search revealed an increasing number of publications and growing academic interest in advanced DWI, the survey revealed limited adoption of advanced DWI techniques among EUSOBI members, with 32% using IVIM models, 17% using non-Gaussian diffusion techniques for kurtosis analysis, and only 8% using DTI. A variety of DWI techniques are used, with IVIM being the most popular, but less than half use it, suggesting that the study identified a gap between the potential benefits of advanced DWI and its actual use in clinical practice.

**Conclusion:**

The findings highlight the need for further research, standardization and simplification to transition advanced DWI from a research tool to regular practice in breast imaging. The study concludes with guidelines and recommendations for future research directions and clinical implementation, emphasizing the importance of interdisciplinary collaboration in this field to improve breast cancer diagnosis and treatment.

**Clinical relevance statement:**

Advanced DWI in breast imaging, while currently in limited clinical use, offers promising improvements in diagnosis, staging, and treatment monitoring, highlighting the need for standardized protocols, accessible software, and collaborative approaches to promote its broader integration into routine clinical practice.

**Key Points:**

- *Increasing number of publications on advanced DWI over the last decade indicates growing research interest*.
- *EUSOBI survey shows that advanced DWI is used primarily in research, not extensively in clinical practice*.
- *More research and standardization are needed to integrate advanced DWI into routine breast imaging practice*.

## Introduction

The concept of diffusion MRI (or diffusion-weighted imaging, DWI) was born in the mid-1980s and has become an essential imaging method in modern diagnostic imaging. Diffusion MRI observes water diffusion in vivo, which is “hindered” by numerous tissue components at a microscopic level, such as cell membranes, fibers, or macromolecules, compared to free water. Therefore, diffusion MRI reflects tissue microstructure and is often used for cancer imaging, as hindrance is usually greater in malignant tissues. For breast imaging, DWI is considered an important addition to dynamic contrast-enhanced MRI (DCE-MRI), especially in terms of improving the specificity of DCE-MRI [1].

The original DWI model relies on a mono-exponential apparent diffusion coefficient (ADC), which integrates all microscopic incoherent motion components (from diffusion, blood flow, etc.) into a single parameter. The ADC concept is mathematically based on a free (Gaussian) diffusion physical framework (as in free water), but inherently also reflects in vivo the hindrance of the molecular displacements by tissue microstructure. In this environment, water diffusion is no longer Gaussian, hence the term “Apparent Diffusion Coefficient” to reflect the fact that the ADC will intrinsically include non-Gaussian diffusion effects (either as a function of diffusion weighting or diffusion time) and deviate from the free diffusion coefficient of water. Non-Gaussian diffusion effects are small at low degrees of diffusion weighting (b-values ≤ 200 s/mm²) and increase with the degree of diffusion weighting, becoming clearly apparent for b-values > 1000 s/mm², which is reflected by the curvature of the log of the signal attenuation versus b-value plot. Hence, the ADC values decrease when the b-value increases. The simplicity of the ADC model and its intrinsic sensitivity to both Gaussian and non-Gaussian diffusion effects have been fundamental to the ability of diffusion MRI to reflect the tissue microenvironment and to its overwhelming success in many clinical applications over the last 40 years [2].

However, more information can be extracted from DWI beyond the ADC [2]. For instance, it is possible to specifically separate Gaussian and non-Gaussian diffusion effects using appropriate representations, such as the Kurtosis approach [3, 4], which might improve clinical performance in lesion classification or staging based on differences in microstructural complexity. On the other hand, other effects with clinical relevance may also be considered, such as blood microcirculation in capillaries which mimics a diffusion process (pseudo-diffusion and intravoxel incoherent motion (IVIM) effect [5]) at very low b-values (≤ 200 s/mm²). Diffusion anisotropy effects (variations of the ADC value depending on the measurement directions in tissues with spatially oriented microstructure) may also be revealed using the Diffusion Tensor Imaging (DTI) framework [6]. In the breast, some anisotropy has been observed in normal fibroglandular tissue, but its clinical relevance for lesion characterization is still a subject of discussion. Also, while most clinical DWI studies have relied on ADC values obtained with intermediate diffusion times (around 50–80 ms), recent technical progress in MRI gradient hardware has made it possible to explore diffusion at either very short or very long diffusion times. When diffusion time increases, diffusive water molecules are able to experience wider environments. Modeling this increase in diffusion hindrance might reveal important information on tissue features (e.g., cell density, cell size or membrane permeability). DWI suffers from distortion and a low signal-to-noise ratio; however, technical advances in MRI gradient hardware and pulse sequence design, as well as higher field strengths and advanced diffusion modeling, have made it possible to overcome the challenges of breast DWI, thereby enabling its broader clinical adoption [7, 8].

In this context, the International Breast DWI group of the European Society of Breast Imaging (EUSOBI) has surveyed its membership on the current clinical usage and the potential of advanced DWI approaches. We present a summary of the survey results and provide some guidance for the next steps required before advanced DWI might be ready for routine usage for breast clinical applications.

## Beyond an “apparent” diffusion coefficient: a short primer of advanced diffusion MRI

The “standard” ADC is obtained as the slope of the logarithm of the DWI signal decay calculated between signals acquired at b0 = 0 and a high b-value, typically b1 = 800 s/mmm^2^ for the breast:

1 $${{{\rm{ADC}}}}={{\mathrm{ln}}}({{{\rm{S}}}}0/{{{\rm{S}}}}1)/({{{\rm{b}}}}1-{{{\rm{b}}}}0)$$

assuming that the signal decay is a straight line in log (signal) vs. b coordinates (mono-exponential model). However, the ADC is reduced when diffusion is not free (non-Gaussian diffusion) in restricted environments, as the signal attenuation becomes curved towards high b-values, and is increased in the presence of blood perfusion, inducing another curvature effect at very low b-values. While the ADC intrinsically encompasses both those effects, the Gaussian and non-Gaussian components of the DWI signal may be more or less sensitive to some features present in tissues, such as intracellular diffusion or extracellular space hindrance, and therefore there is a diagnostic motivation to resolve them.

### At low b-values: IVIM

The DWI signal reflects not only molecular diffusivity in tissues but also microcapillary flow, which mimics a pseudo-diffusion process due to the quasi-random organization in space of capillary segments. The classical bi-exponential IVIM model enables the separation of the intravoxel signal into molecular diffusion and microcirculation (perfusion). It was initially introduced and developed by Le Bihan et al to quantitatively assess all the microscopic translational motions contributing to the signal changes measured by diffusion MRI [9]. In this model, perfusion-free molecular diffusion (D or Dt), perfusion-related pseudo-diffusion (D*, Dp, or Df) and flowing blood fraction (perfusion) (f, fp, or f_IVIM_) are calculated by

2 $${{{\rm{S}}}}({{{\rm{b}}}})={{{\rm{S}}}}(0)[(1-{{{\rm{f}}}})\exp  (-{{{\rm{bD}}}})+{{{\rm{f}}}}\,\exp  (-{{{\rm{bD}}}}^{\ast} )]$$

where S(b) is the signal intensity at a given b-value, and S0 is the signal intensity in the absence of diffusion weighting.

In practice, IVIM and diffusion parameters are often estimated using a two-step approach, first fitting the data signals with the diffusion part of Eq. [2] to get D, which implies choosing a b-value threshold above which IVIM effects are negligible. Once D is estimated, f and D* are estimated by fitting data signals at all b-values with Eq. [2]. In the breast, the threshold is often between 200 and 400 s/mm^2^ [10] depending on the tissue f and D* values. However, there is no consensus yet on the number of b-values that should be acquired below and above this threshold. The more the number of b-values, the more accurate the parameter estimates, but at the price of an increase in acquisition time.

### At high b-values: non-Gaussian DWI

On the other extreme, deviation from a mono-exponential (Gaussian) signal behavior at high b-values might inform on the complexity of tissues. Non-Gaussian diffusion parameters may thus be regarded as markers of tissue microstructure. Among non-Gaussian diffusion models, diffusion kurtosis imaging (DKI) is a widely used mathematical non-Gaussian model [3, 4], with ADCo, which represents true Gaussian diffusion (as would be obtained when b becomes close to 0 in the absence of perfusion-driven IVIM effects) and K, the kurtosis parameter which represents the deviation from Gaussian diffusion, given by:

3 $${{{\rm{S}}}}(b)={{{\rm{S}}}}(0) \exp  (-{{{\rm{b}}}} {{{\rm{ADC}}}}_{0}+{({{{\rm{b}}}}{{{\rm{ADC}}}}_{0})}^{{2}} {{{\rm{K}}}}/6)$$

**Fig. 1:**
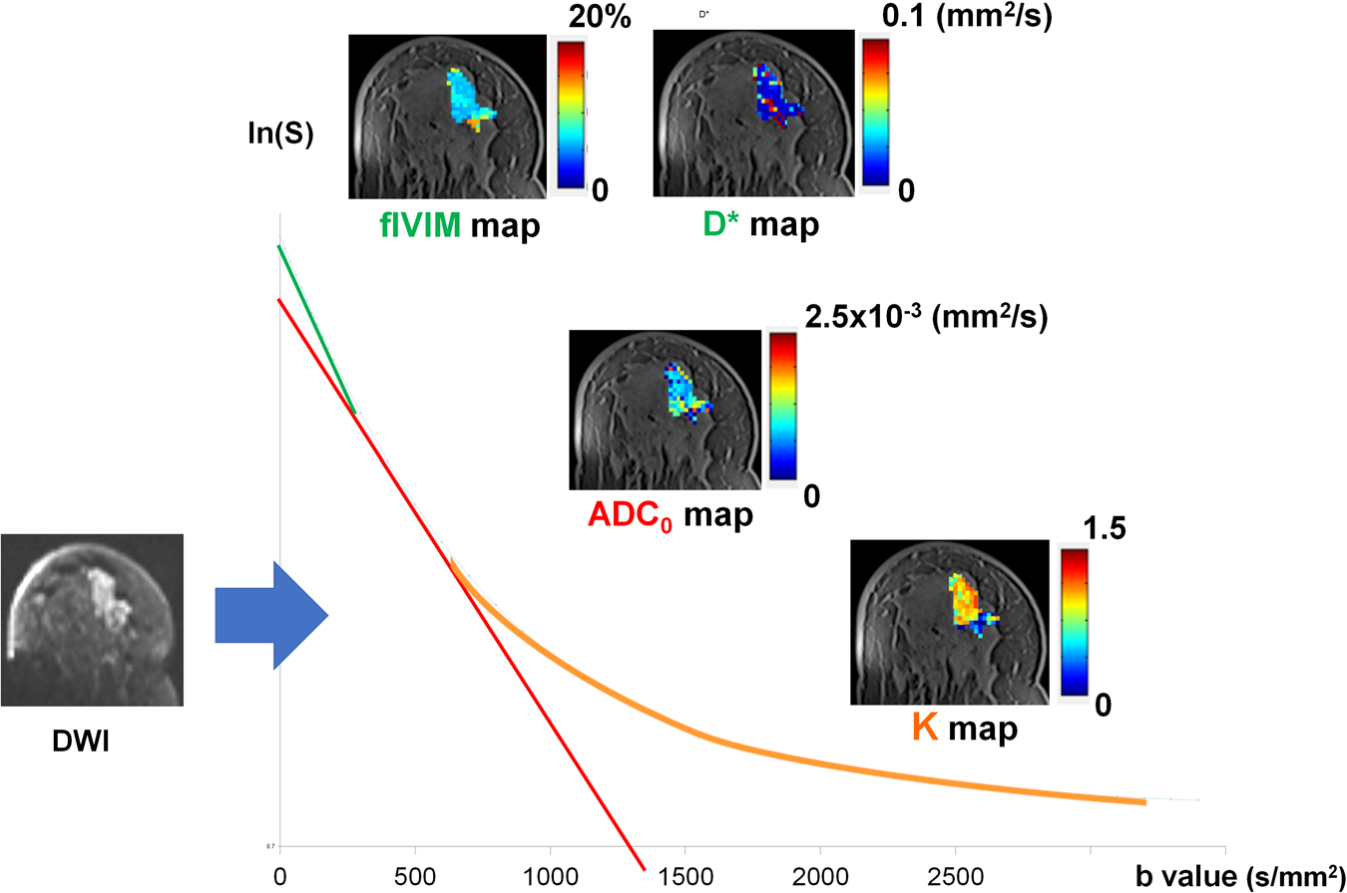
Advanced DWI maps and DWI signal decay versus b-values of breast cancer. Beyond conventional ADC map, new advanced DWI (IVIM (f, fp or f_IVIM_, and D*), non-Gaussian (ADCo, K)) maps can be extracted by analyzing DWI maps acquired at many b-values (courtesy of the breast imaging group at the Radiology department of Kyoto University)

These advanced DWI maps and DWI signal decay versus b-values are graphed in Fig. 1. It is also necessary to keep in mind that the use of trace-weighted images in the presence of diffusion anisotropy may introduce non-negligible errors and biases in the estimation of diffusion kurtosis imaging-derived indices of the breast [11, 12]. These biases can be alleviated by the collection of more diffusion directions (minimally 15 for kurtosis tensor determination), but this must be considered in balance with the longer associated scan time.

The stretched exponential model is a modified form of the mono-exponential equation by using a distributed diffusion coefficient (DDC), given by:

4 $${{{\rm{S}}}}({{{\rm{b}}}})={{{\rm{S(0)}}}} \exp {(-{{{\rm{b}}}} {{{\rm{DDC}}}})}^{{{{\rm{\alpha }}}}}$$

where DDC represents a summary of the distribution of the diffusion coefficients in the voxel and α represents a heterogeneity index.

It should be noted that such models while quantifying the degree of non-Gaussian diffusion, do not provide any information on the mechanisms (cell density, cell membranes, fibers, membrane permeability, etc.). The earliest and simplest attempt to provide such specificity may be a two-compartment model with a fast and a slow diffusion component [13]. However, this approach alone neglects membrane permeability and compartmental relaxation effects so that the precise interpretation of the observed compartments remains elusive. More biophysically explicit models have been proposed to provide such information (vascular, extracellular and restricted diffusion for cytometry in tumors (VERDICT) [14], imaging microstructural parameters using limited spectrally edited diffusion (IMPULSED) [15]). While developed originally to investigate brain tissue (both gray and white matter), they have since been applied in other contexts, including cancer (e.g., a recent review [16]). While such development for breast tissue remains in its infancy, some pilot studies have already been performed [17–19].

### Combined IVIM and non-Gaussian diffusion

As IVIM effects are present concurrently with non-Gaussian diffusion effects in many tissues, including cancerous tissue, it is natural to combine the two approaches. With the IVIM/Kurtosis model, one has to fit DWI signals with the following equation:

5 $${{{\rm{S}}}}({\rm{b}})={{{\rm{S}}}}(0)[f{\cdot}exp \, (-b D^* )+(1-f) exp \, (-bADC_{0}+(bADC_{0})^2K/6)]$$

Furthermore, dedicated signal processing strategies must be implemented to obtain parameter estimates with good accuracy. Most often, this involves a two-step process: first for microstructural diffusion metrics and then for perfusion-driven IVIM. However, there are other possible approaches, such as Bayesian analysis [20], the use of libraries of synthetic signals generated by ranges of parameters [2], and deep learning [21, 22].

A drawback of the combined model is that the high number of parameters that have to be estimated (there is only one for the ADC) requires the acquisition of a number of signals at different b-values (at least one per parameter, but often many more to provide sufficiently high precision), leading to long acquisition times (around more than 5 min depending on the number of b-values) difficult to impose in a clinical environment. With the combined IVIM/Non-Gaussian diffusion model, often as many as 12–16 signals are collected on a wide range of b-values between 0 and 1500 or even 2500 s/mm², though this would result in long acquisition times. An abbreviated protocol based on four key b-values has recently been proposed to evaluate f, ADCo, and K (50).

### Orientation effects and diffusion anisotropy

Diffusion tensor imaging (DTI) provides a more comprehensive directional characterization of tissue by measuring diffusion across multiple directions, capturing both average diffusion coefficients and anisotropy. DTI calculates the mean diffusion,

6 $${{{\rm{MD}}}}=({{{\rm{\lambda }}}}1+{{{\rm{\lambda }}}}2+{{{\rm{\lambda }}}}3)/3$$

and fractional anisotropy (FA) using eigenvalues, calculated as

7 $${FA}=\sqrt{\left(\frac{1}{2}\right)* \frac{\left[{\left({{{\rm{\lambda }}}}1-{{{\rm{\lambda }}}}2\right)}^{2}+\,{\left({{{\rm{\lambda }}}}2-{{{\rm{\lambda }}}}3\right)}^{2}+\,{\left({{{\rm{\lambda }}}}3-{{{\rm{\lambda }}}}1\right)}^{2}\right]}{{{{\rm{\lambda }}}}{1}^{2}+{{{\rm{\lambda }}}}{2}^{2}+{{{\rm{\lambda }}}}{3}^{2}}}$$

which represent diffusion magnitudes along orthogonal principal axes(‘eigenvectors’) that indicate the principal orientation of tissue microstructure. DTI and its variants have been and continue to be extensively used in neuroimaging for the characterization of anisotropic diffusion in white matter axons [23–26]. Analogously, many studies have shown anisotropic diffusion in breast fibroglandular tissue (FGT) in comparison with more disordered lesions [7, 8, 27]. However, its non-linear and noise-sensitive nature requires careful interpretation. Noise can lead to misleading anisotropy indications (“eigenvalue repulsion”), which can artificially enhance the distinction of malignancy from FGT (see more detail in [28]). Thus, as with all advanced methods, sufficient SNR must be assured to support the higher order signal analysis and associated biomarker generation.

### Effects of the diffusion time

Diffusion time is also an important factor in DWI contrast. ADC values decrease proportional to an increase in diffusion time, as water molecules have a greater probability of interacting with tissue microstructures. Stimulated echo DWI pulse sequences [29] achieve long diffusion times up to 1 s, which sensitizes the signal to larger structures such as the radial length scale of ducts. Conversely, oscillating gradient spin-echo (OGSE) can achieve DWI with short diffusion times (a few ms) by oscillating motion probing gradient into a waveform or trapezoidal form. These short diffusion times provide access to correspondingly small-length scales of cancerous cellularity (a few microns).

## Current status on the clinical application of advanced DWI models to the breast: narrative survey of the EUSOBI International Breast Diffusion-weighted Imaging working group

### Survey design

One radiologist and doctoral imaging scientist certified with over a decade of experience in breast imaging, and breast MRI in particular, designed a questionnaire consisting of 20 questions related to technical preferences of advanced DWI (see Electronic Supplementary Material). Following approval by the EUSOBI executive board, the questionnaire was made accessible through an online platform (Google Forms). Radiologists who were active members of EUSOBI or associated members not based in Europe received anonymous invitation emails from the central EUSOBI office on January 27, 2020, which included a link to the questionnaire in the email body. This narrative survey delves into the utilization of advanced breast DWI within the EUSOBI community, based on a comprehensive review of accumulating evidence. While advanced breast DWI has shown promise in clinical research, its integration into clinical practice remains limited. The findings are summarized below, followed by some guidelines and recommendations for its future application.

### Literature search

**Fig. 2:**
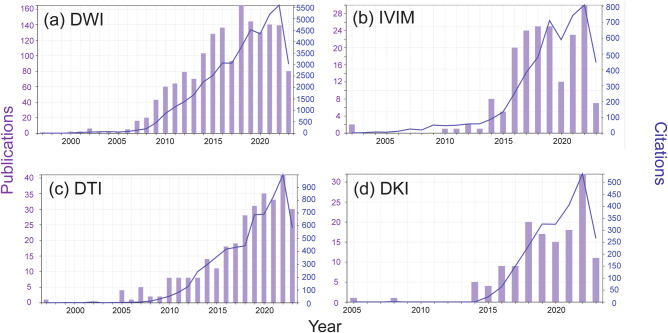
Publication and citation timeline for applications of DWI techniques to the breast. **a** Diffusion-weighted imaging; **b** Intravoxel incoherent motion (IVIM); **c** Diffusion tensor imaging (DTI); **d** Diffusion kurtosis imaging (DKI)

The search terms are detailed in the Electronic Supplementary Material. Figure 2 and Table 1 depict these trends by showing the annual publications and citations, as well as their total and average h-index in each sub-category. While the advanced approaches occupy a smaller footprint of the literature compared to conventional DWI by as much as a factor of 10, it is evident that they are experiencing a significant increase in publication activity, reflecting a substantial interest in this topic within the research community, particularly in the last decade.

**Table 1:** Summary of publication record for diffusion MRI variants as applied to breast imaging (Source: ISI Web of Science)

Topic	Publications	Citations	H-index
DWI	1631	43,446	92
IVIM	186	4966	34
DTI	307	6291	42
DKI	142	2319	25

### Survey results

Of 1287 EUSOBI radiologist members, 25/1287 (2%) responded to the survey.

### Usage patterns

Among the surveyed respondents, advanced breast DWI has predominantly been employed for clinical research purposes, with limited adoption in clinical practice. Notably, 32% of the participants (8/25) employ the IVIM model, with half favoring the IVIM/Gaussian model and the other half opting for the IVIM/non-Gaussian model. The data processing approaches vary, with 71% (5/7) relying on segmented fitting in two steps using the chosen IVIM models. Non-Gaussian diffusion techniques, particularly for Kurtosis analysis, are utilized by 17% (4/23) of respondents.

**Fig. 3:**
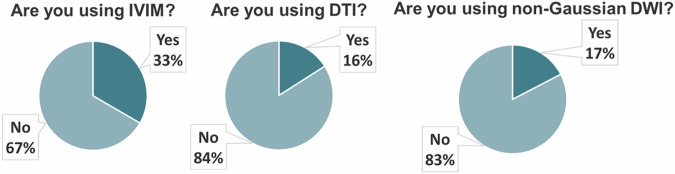
A pie chart depicting the percentage of EUSOBI survey respondents (*n* = 25) who responded affirmatively to using intravoxel incoherent motion (IVIM), diffusion tensor imaging (DTI), and non-Gaussian DWI, respectively

**Fig. 4:**
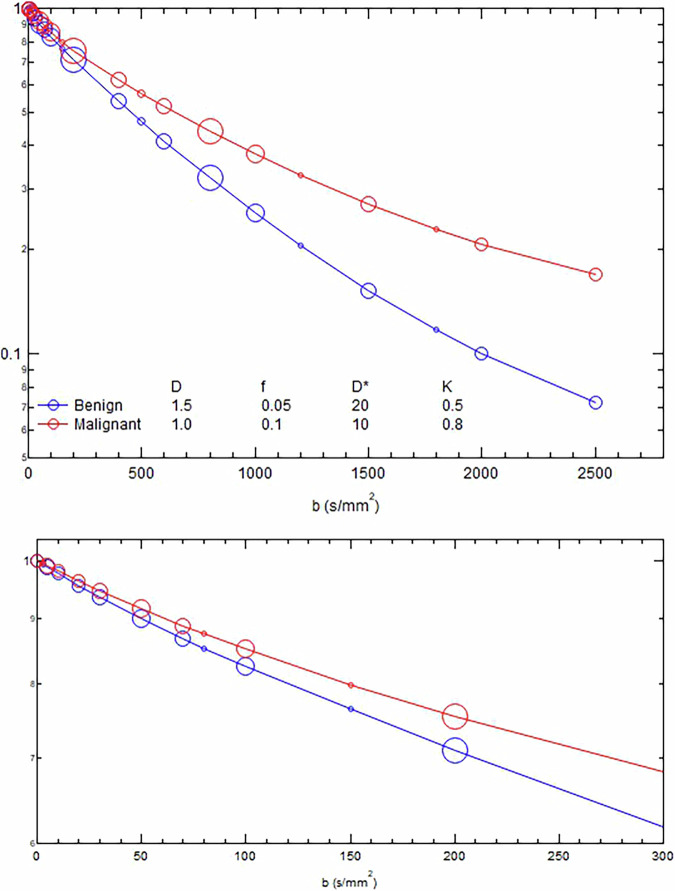
Usage of b-values in DWI decay curves for typical benign/malignant lesions (point size proportional to frequency of usage) from participants in the survey

A small subset (8%, 4/25) reported using DTI with standard parameters such as MD, FA, and λ values. Figure 3 shows the graph illustrating the proportion of EUSOBI members who indicated they utilize IVIM, DTI, and non-Gaussian DWI, respectively. Although IVIM techniques are the most frequently mentioned, they are used by less than half of the respondents, and they have not yet found widespread clinical application. Based on these results, the path to the clinical implementation of advanced DWIs may still be a long journey. The usage of b-values is summarized in Fig. 4.

### Discussion and limitations

This survey clearly shows that advanced diffusion MRI is not yet widely used in clinical practice. The adoption of advanced breast DWI remains primarily within the realm of research, indicating a gap between its potential and clinical integration. However, only a small number of responses were received to the survey, and the present results have little impact. This limitation is because advanced DWI is not widespread among EUSOBI members currently. In the next section, we are addressing key questions that might hamper the clinical implementation of advanced diffusion MRI. This survey has not considered some aspects of practical interest: diffusion gradient non-linearity, eddy currents correction, and related geometric distortion.

## Obstacles to be removed for the use of advanced DWI models to the breast

### Clinical performance: what are the benefits of advanced DWI compared to standard DWI

#### IVIM

IVIM helps distinguish malignant from benign breast lesions based on higher perfusion fraction (f) and lower diffusion coefficient (D) in malignancies, and its AUC (0.85 for f and 0.91 for D) is comparable to or higher than ADC (AUC = 0.85) [30]. IVIM can improve breast cancer diagnostic accuracy when combined with DCE-MRI [31–35]. Lower D values are associated with more invasive and aggressive breast cancers, such as invasive ductal carcinoma and Ki-67 overexpressing cancers [30, 36]. D was also reported to be the most useful parameter in predicting axillary lymph node metastasis [37]. Additionally, a higher pre-treatment D value and its change post-treatment are indicative of a favorable response to neoadjuvant systemic therapy [38, 39].

#### DKI

DKI metrics MD and MK effectively distinguish between malignant and benign breast lesions (AUC = 0.91 and 0.95) [40]. While correlations between DKI parameters and cancer markers like Ki-67 are varied [41, 42], higher K values may suggest a greater risk of metastasis [43]. DKI is also useful in analyzing microstructural changes in triple-negative breast cancer [44].

#### Stretched exponential model

DDC and α values, estimated using the stretched exponential model, were shown to be lower in malignant tumors than in normal breast tissues and benign tumors [45]. Suo et al showed that DDC was lower in ER-positive vs. ER-negative cancers, and α was lower in high vs. low Ki-67 cancers [46].

#### DTI

Cancerous tissue can reduce anisotropy by blocking or disrupting the glandular microarchitecture. Lower λ1 and lower MD are usually associated with malignancy (AUC = 0.97, 0.92 and 0.76) [47], in line with the overall decrease in ADC in malignant lesions. Reduced (λ1 – λ3) values (or FA) have sometimes been reported. However, this does not necessarily mean reduced anisotropy, as DTI is non-linearly sensitive to background noise, making low ADC tissues artificially appear with less differences in λ1 and λ3values [25]. DTI parameters in normal breast tissue are consistent throughout the menstrual cycle, with diagnostic accuracy likely unaffected by timing. While diffusivity differs between pre- and post-menopausal women, anisotropy does not. In lactating breasts, a study suggested that DTI could be more effective than DCE-MRI for tumor detection, due to reduced lesion visibility from marked background parenchymal enhancement on DCE-MRI [48].

#### Diffusion time

ADC, with a short diffusion time, might capture the movement of water molecules on a cellular scale. Short and long diffusion time ADC measurements were demonstrated to distinguish between different types of breast cancer xenografts [49] or different cellular compositions and sizes [50].

DTI with long diffusion times using stimulated-echo acquisition mode (STEAM) was reported to be sensitive to large-scale structures in fibroglandular tissue [51].

#### Summary

As mentioned above, some studies have demonstrated a comparable or higher diagnostic ability of advanced DWI than ADC in distinguishing malignant from benign breast lesions, but the evidence is currently not enough to determine whether a biopsy is necessary based only on these advanced DWI techniques. Regarding the prediction of prognostic factors, it is still inconclusive whether the advanced MRI outperforms conventional ADC. Characterization of small foci using these advanced DWI techniques might be difficult, considering the resolution and noise effects. Compared to conventional ADC, advanced DWI has the potential to capture information about breast cancer that has not been captured before, rather than improving discrimination between malignant from benign breast lesions. Some studies have shown that these parameters predict distant metastasis, which is important for determining clinical management and highly encouraging.

### Implementation on clinical scanners

#### Recommendations

Further research is encouraged to establish robust protocols and parameter guidelines specific to advanced breast DWI. Standardization of acquisition and analysis, as well as the growing application of standardized phantoms for breast DWI [52], will aid in improving the reproducibility and comparability of results. While standardization facilitates data pooling and multicentric studies, it is equally important to ensure that these standardized methods are optimized for reliable and accurate quantitative diffusion measurements. Collaborative efforts among researchers, clinicians, and industry experts, including consulting with a medical physicist specializing in MRI, are crucial to develop user-friendly analysis software that bridges the gap between research and clinical applications.

Advanced DWI models are challenged by long acquisition times, prompting research into simplified approaches. One method involves estimating IVIM and non-Gaussian parameters using just four b-values [53]. Another approach generates “synthetic” indices like sADC (shifted ADC) [54] (b0 = 200 s/mm² and b1 = 1500 s/mm², for instance) and S-index from a limited set of b-values, focusing more on non-Gaussian diffusion. These indices, although less biologically specific, show promise in classifying breast lesions and estimating prognostic biomarkers [55, 56]. They offer a potential compromise for certain clinical tasks like screening or subtyping, balancing efficiency and diagnostic value.

The workload of radiologists may not be affected as much, because the results can be quantitatively evaluated and displayed on color maps. If diffusion MRI becomes a substitute (instead of an add-on) to contrast-enhanced MRI hopefully in the future, both scanning and reading times will be saved.

### Technical advances

While recent technical advances clearly benefit standard DWI, for instance, by increasing spatial resolution and decreasing the occurrence of artifacts, the gain should similarly apply to advanced DWI approaches, and thus, some of these efforts are summarized below.

## Fat suppression

Fat suppression is essential for DWI but even more for advanced diffusion MRI, as the insufficient suppression of a high signal in fat may lead to errors in parameter estimations, such as ADC underestimation. In addition, fat contains multiple peaks, and the effective suppression of all fat peaks remains challenging. Conventional fat suppression includes spectral attenuated inversion recovery (SPAIR) and short tau inversion recovery (STIR). EUSOBI currently recommends the use of SPAIR in acquiring breast DWI with the evaluation of ADC given a moderate preference in literature in comparison with STIR [1]. The combination of water-excitation and spectral fat saturation has recently been shown to provide superior signal-to-noise, contrast-to-noise and signal-intensity ratios for breast lesions compared to standard SPAIR [57].

## Image quality, spatial resolution and geometric image distortion

Other important issues are spatial resolution, not only to allow detection of small lesions, but also to decrease partial volume averaging effects (resulting in less intravoxel inhomogeneity), hence more suitable to the available DWI models, and geometric distortion, as models require the combination of a set of images acquired with different b-values, hence different levels of distortion.

**Fig. 5:**
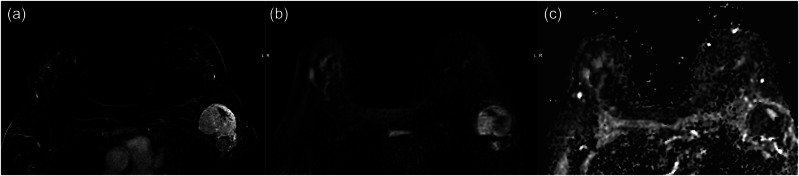
Triple-negative invasive ductal carcinoma upper outer quadrant left breast posterior depth. Contrast-enhanced image (**a**), DW image (**b**), and ADC map (**c**). The multiplexed sensitivity encoding (MUSE) DW images were obtained using the deep learning reconstruction technique from GE healthcare. The tumor is clearly delineated, and detailed intratumor heterogeneity is preserved in the deep learning DW image. The use of deep learning reconstruction not only facilitates a potential reduction in scan time but also improvement in image quality

Fat-suppressed single-shot echo-planar imaging (ss-EPI) has been the standard for breast DWI, valued for its rapid readout that minimizes motion artifacts and its widespread availability. However, ss-EPI suffers from distortion and blurring, leading to the development of other techniques like multi-shot readout segmented EPI DWI (RESOLVE) [58, 59] and multiplexed sensitivity encoding (MUSE), shot-locally low rank (LLR), and simultaneous multislice (SMS) to improve image quality [60, 61]. RESOLVE and MUSE reduce distortion, LLR offers higher resolution and less ghosting, and SMS accelerates scan time while maintaining or enhancing image quality. Studies have shown that advanced methods like SMS can provide superior image quality and lesion characterization compared to ss-EPI [62]. Figure 5 shows representative MUSE DW images reconstructed with the deep learning technique of breast cancer. Additionally, techniques like SPEN [63]. and reduced FOV methods like ZOOMit [64] and FOCUS further enhance image quality by minimizing distortion and foldover artifacts. These advancements allow for more effective differentiation of benign and malignant breast lesions, with improved spatial resolution and detailed tumor characterization [65]. Future improvements would focus on addressing diffusion gradient non-linearity, eddy currents and geometric distortion correction which could be a relevant source of inaccuracy in voxelwise estimation of quantitative diffusion indices [66], and are often overlooked in breast DWI research studies. Additionally, it is important to assess any miscalibration [67] or excessive non-linearity degree [68] of diffusion weighting gradients to obtain a reliable estimation of quantitative diffusion indices.

## Conclusion

In summary, the EUSOBI survey on advanced breast DWI highlights its potential but limited clinical use, mostly in research. The survey emphasizes the need for standardized protocols and user-friendly software for clinical applications. Advanced DWI techniques offer potential benefits in lesion characterization, staging, and treatment response prediction. As the field progresses, interdisciplinary collaboration between radiologists, physics and vendors, as well as cross-discipline conferences between radiologists and oncologists, will be instrumental in refining protocols and promoting broader adoption in routine clinical practice. The insights gained from this survey will guide future research and efforts toward enhancing breast cancer diagnosis and patient care.

## Supplementary information

ELECTRONIC SUPPLEMENTARY MATERIAL

## Abbreviations

ADC: Apparent diffusion coefficient
BI-RADS: Breast Imaging Data and Reporting System
DCE-MRI: Dynamic contrast-enhanced magnetic resonance imaging
DWI: Diffusion-weighted imaging
EUSOBI: European Society of Breast Imaging
