# Supplementary material for: Advanced breast diffusion-weighted imaging: what are the next steps? A proposal from the EUSOBI International Breast Diffusion-weighted Imaging working group
Source: Eur Radiol. 2024 Oct 8;35(4):2130–40. doi: 10.1007/s00330-024-11010-0 (PMC11914331; doi:10.1007/s00330-024-11010-0)
Supplement: Supplementary file 1 — ELECTRONIC SUPPLEMENTARY MATERIAL [file 330_2024_11010_MOESM1_ESM.pdf]

Electronic Supplementary Material

A questionnaire consisting of 20 questions related to technical preferences of advanced DWI

IVIM

Are you using IVIM?

NO→please go to DTI section

What is your protocol?

Field strength

RF coil (number of channels)

b values

number of directions for diffusion encoding (ex 1, 3, 3in1, 6,etc)

'type of sequence' (ex diffusion EPI)

number of excitations or averages

TE(ms)

TR(ms)

voxel dimensions in mm (ex 3x3x3)

image orientation

volume of sampling (single slice, whole lesion, whole breast)

Other comments

Which method have you been using for IVIM analysis?

Fitting one-step approach (biexponential, nonlinear least-squares)

Fitting two-step approach (segmented)

Bayesian

Non-negative least-squares

Dictionary-based method

Neural Networks

Non-fitting: RED (Relative Enhanced Diffusivity) (different from IVIM, but one can estimate microperfusion from DWI)

denoising stage?

Other

Do you use associated DWI model?

Gaussian or IVIM biexponential

IVIM + non-Gaussian diffusion

Which output or parameters do you obtain?

f,fp,fIVIM

D*,Dp

D, Dt

other

Which software are you using?

in house software

→please indicate platform (matlab etc)

commercial software

→please indicate vendor (Olea etc)

other

Have you used those results in clinical practice?

- usage: research / clinical research / clinical practice

DTI

Are you using DTI?

NO→please go to non-Gaussian dffusion section

What is the protocol?

- Field strength
- RF coil (number of channels)
- b values (shells)
- number of directions for diffusion encoding (ex 1, 3, 3in1, 6,etc)
- 'type of sequence' (ex diffusion EPI)
- number of excitations or averages
- TE(ms)
- TR(ms)
- voxel dimensions in mm (ex 3x3x3)
- image orientation
- volume of sampling (single slice, whole lesion, whole breast)
- Other comments

Which method have you been using for DTI analysis?

- Direct calculation
- Non-linear fitting
- Multiple linear regression analysis
- denoising stage?

Combined with IVIM

yes/no

Which output or parameters do you obtain?

- MD
- FA
- $\lambda 1, \lambda 2, \lambda 3$
- e1, e2, e3
- Dr (radial diffusivity)
- Da (axial diffusivity)
- Tractography

Cl, Cp, Cs (linear / planar / spherical components)

Which software are you using?

in house software

→please indicate platform (matlab etc)

commercial software

→please indicate vendor (Olea etc)

other

Have you used those results in clinical practice?

- usage: research / clinical research / clinical practice

Non-Gaussian Diffusion

Are you investigating non-Gaussian diffusion?

NO→please go to special sequences section

What is the protocol?

- Field strength
- RF coil (number of channels)
- b values
- number of directions for diffusion encoding (ex 1, 3, 3in1, 6,etc)
- 'type of sequence' (ex diffusion EPI)
- number of excitations or averages
- TE(ms)
- TR(ms)
- voxel dimensions in mm (ex 3x3x3)
- image orientation
- volume of sampling (single slice, whole lesion, whole breast)
- Other comments

Which model have you been using for the analysis?

- Kurtosis
- Stretched exponential
- Biexponential
- Hybrid IVIM / non-gaussian fit

Which method have you been using for the analysis?

- Least-squares Fitting
- Bayesian
- Non-negative least-squares
- Dictionary-based method
- Neural Networks
- denoising stage?

Which output or parameters do you obtain?

- K
- MD

alpha

D1, D2, f1, f2

other

Which software are you using?

in house software

→please indicate platform (matlab etc)

commercial software

→please indicate vendor (Olea etc)

other

Have you used those results in clinical practice?

- usage: research / clinical research / clinical practice

Literature search

A public tool (Web of Science) was utilized to summarize the publication history of techniques in the realm of diffusion MRI as applied to breast imaging, with the following search terms: “Breast” AND (1) “Diffusion weighted imaging”, (2) “IVIM or intravoxel incoherent motion”, (3) “DTI or diffusion tensor imaging”, or (4) “DKI or diffusion kurtosis imaging”
